# The high-sensitivity modified Glasgow prognostic score is superior to the modified Glasgow prognostic score as a prognostic predictor for head and neck cancer

**DOI:** 10.18632/oncotarget.26438

**Published:** 2018-12-11

**Authors:** Nobuhiro Hanai, Michi Sawabe, Takahiro Kimura, Hidenori Suzuki, Taijiro Ozawa, Hitoshi Hirakawa, Yujiro Fukuda, Yasuhisa Hasegawa

**Affiliations:** ^1^ Department of Head and Neck Surgery, Aichi Cancer Center Hospital, Aichi, Japan; ^2^ Department of Otorhinolaryngology, Head and Neck Surgery, Nagoya City University Graduate School of Medical Sciences, Aichi, Japan; ^3^ Department of Otolaryngology-Head and Neck Surgery, Nara Medical University, Nara, Japan; ^4^ Department of Otolaryngology, Toyohashi Municipal Hospital, Aichi, Japan; ^5^ Department of Otorhinolaryngology, Head and Neck Surgery, Graduate School of Medicine, University of the Ryukyus, Okinawa, Japan; ^6^ Department of Otolaryngology, Kawasaki Medical School, Okayama, Japan; ^7^ Department of Head and Neck Surgery and Otolaryngology, Asahi University Hospital, Gifu, Japan

**Keywords:** modified Glasgow prognostic score, high-sensitivity modified Glasgow prognostic score, head and neck cancer, C-reactive protein, survival

## Abstract

**Background:**

There is increasing evidence that the inflammatory indices of modified Glasgow prognostic score (mGPS) and high-sensitivity mGPS (HS-mGPS) play important roles in predicting the survival in many cancer; however, evidence supporting such an association in head and neck cancer (HNC) is scarce.

**Materials and Methods:**

We evaluated the impact of the mGPS and HS-mGPS on the overall survival (OS) in 129 patients with HNC treated at Aichi Cancer Center Central Hospital from 2012-2013. The mGPS was calculated as follows: mGPS of 0, C-reactive protein (CRP) ≤1.0 mg/dl; 1, CRP >1.0 mg/dl; 2, CRP>1.0 mg/dl and albumin <3.5 mg/dl. Regarding the HS-mGPS, the CRP threshold level was set as 0.3 mg/dl. Hazard ratios (HRs) and 95% confidence intervals (95% CIs) were estimated by Cox proportional hazard models after adjusting for potential confounders.

**Results:**

The prognosis of HNC worsened significantly as both the mGPS and HS-mGPS increased in a univariate analysis. After adjusting for covariates, the HS-mGPS was significantly associated with the OS (adjusted HR for HS-mGPS of 2 compared to an HS-mGPS of 0 [HR_score2-0_] 3.14 [95% CI: 1.23-8.07], P_trend_ < 0.001), while the mGPS was suggested to be associated with the survival (HR_score2-0_ 2.37 [95% CI:0.89-6.33], P_trend_ = 0.145). Even after stratification by clinical covariates, these associations persisted.

**Conclusion:**

We conclude that the HS-mGPS is useful as an independent prognostic factor in HNC.

## INTRODUCTION

The definition of cancer cachexia according to the 2011 European Palliative Care Research Collaborative (EPCRC) guidelines is a state of progressive malnutrition due to hypercatabolism, which can occur due to metabolic disorders [1]. It is associated with the destruction of skeletal muscle by systemic inflammatory reactions. The state gradually becomes irreversible, even if the nutritional status is restored. Thus, it is difficult to improve cancer cachexia by conventional nutritional support, and providing nutritional support from an early stage is considered important [2].

The Glasgow Prognostic Score (GPS) has been reported to be a useful inflammatory index for assessing the status of cachexia [3]. This score is composed of C-reactive protein (CRP) to reflect the systemic inflammation status and serum albumin levels to reflect the nutrition status [4]. At present, the modified GPS (mGPS) is widely used to classify patients into three groups: mGPS=0, 1, 2 as shown in Table 1. The correlation between the mGPS and the prognosis has been proven in gastroenterological cancers (colorectal cancer [5, 6], gastric cancer [7–9]) as well as in lung cancer [10, 11] and urological cancer [12]. Furthermore, the recently established high-sensitivity modified Glasgow prognostic score (HS-mGPS) is considered to be an even more sensitive prognostic marker for those cancers [13–16].

**Table 1:** Criteria of systeminc inflammation-based prognostic scores, mGPS and HS-mGPS

Prognostic score	Criteria	Score allocated
mGPS	CRP ≤1.0mg/dl	0
	CRP>1.0mg/dl and Alb≧3.5g/dl	1
	CRP>1.0mg/dl and Alb<3.5g/dl	2
HS-mGPS	CRP≦0.3mg/dl	0
	CRP>0.3mg/dl and Alb≧3.5g/dl	1
	CRP>0.3mg/dl and Alb<3.5g/dl	2

mGPS, Modified Glasgow prognostic score; HS-mGPS, High-sensitivity modified Glasgow prognostic score; CRP, C-reactive protein; Alb, Albumin.

Head and neck cancer (HNC) often causes symptoms associated with deglutition, suggesting that many of these patients might be suffering from undernutrition [17], which can lead to cachexia. Therefore, the prognostic impact of the mGPS with regard to the status of cancer cachexia in HNC should be explored. However, to our knowledge, there have been only two reports evaluating the association between the mGPS and the prognosis of HNC [18, 19]. Furthermore, evidence concerning the influence of HS-mGPS, which may be more sensitive than the mGPS for assessing the state of cachexia, on the survival impact is lacking.

This retrospective cohort study therefore explored whether or not the mGPS/HS-mGPS has prognostic utility and evaluated which is superior for predicting the prognosis of HNC in a Japanese population.

## RESULTS

### Patient characteristics

The median follow-up period was 1308 days (range: 118-1580 days). During the follow-up period, 15 patients were lost to follow-up. The demographic characteristics of the patients are shown in Table 2. The median age was 65 years (range: 23-84 years). One hundred and five of the 129 (81.4%) patients were men, and 24 (18.6%) were women. The majority of the patients had a PS of 0 (62.8%) and stage IV disease (62.0%). The distribution of the primary tumor was as follows: nasal cavity and paranasal sinuses (n=12), oral cavity (n=35), oropharynx (n=29), hypopharynx (n=37) and the larynx (n=16). Ten patients (8%) were found to have multiple primary cancer (MPC), half of whom had esophageal cancer and the other half aerodigestive tract cancer.

**Table 2:** Patient characteristics

Characteristics	*N* (%)	mGPS				HS-mGPS			
		0	1	2	p-value^3)^	0	1	2	p-value^3)^
Age									
<65 years	61 (47.3)	53	6	2	0.177	43	13	5	0.140
≥65 years	68 (52.7)	51	10	7		36	23	9	
Sex									
male	105 (81.4)	83	16	6	0.020	62	33	10	0.118
female	24(18.6)	21	0	3		17	3	4	
PS^1)^									
0	81 (62.8)	76	6	1	<0.001	61	16	4	<0.001
1	40 (31.0)	26	10	4		16	18	6	
2	7 (5.4)	4	0	3		2	2	3	
3	1 (0.8)	0	0	1		0	0	1	
Stage^2)^									
I	6 (4.7)	6	0	0	0.119	5	1	0	0.001
II	23 (17.8)	21	2	0		21	2	0	
III	20 (15.5)	18	1	1		15	3	2	
IV	80 (62.0)	59	13	8		38	30	12	
Primary site									
nasal cavity	12 (9.3)	7	4	1	0.669	3	8	1	0.183
oral cavity	35 (27.1)	30	3	2		26	6	3	
oropharynx	29 (22.5)	23	5	1		16	9	4	
hypopharynx	37 (28.7)	31	3	3		25	9	3	
larynx	16 (12.4)	13	1	2		9	4	3	
Multiple primary cancer					0.532				0.417
Present	10 (7.8)	8	2	0		6	4	0	
Absent	119 (92.3)	96	14	9		73	32	14	

1)Eastern Cooperative Oncology Group performance status (ECOG PS).

2)UICC 7th edition.

3)Chi-square test or Fisher's exact test.

**Figure 1:**
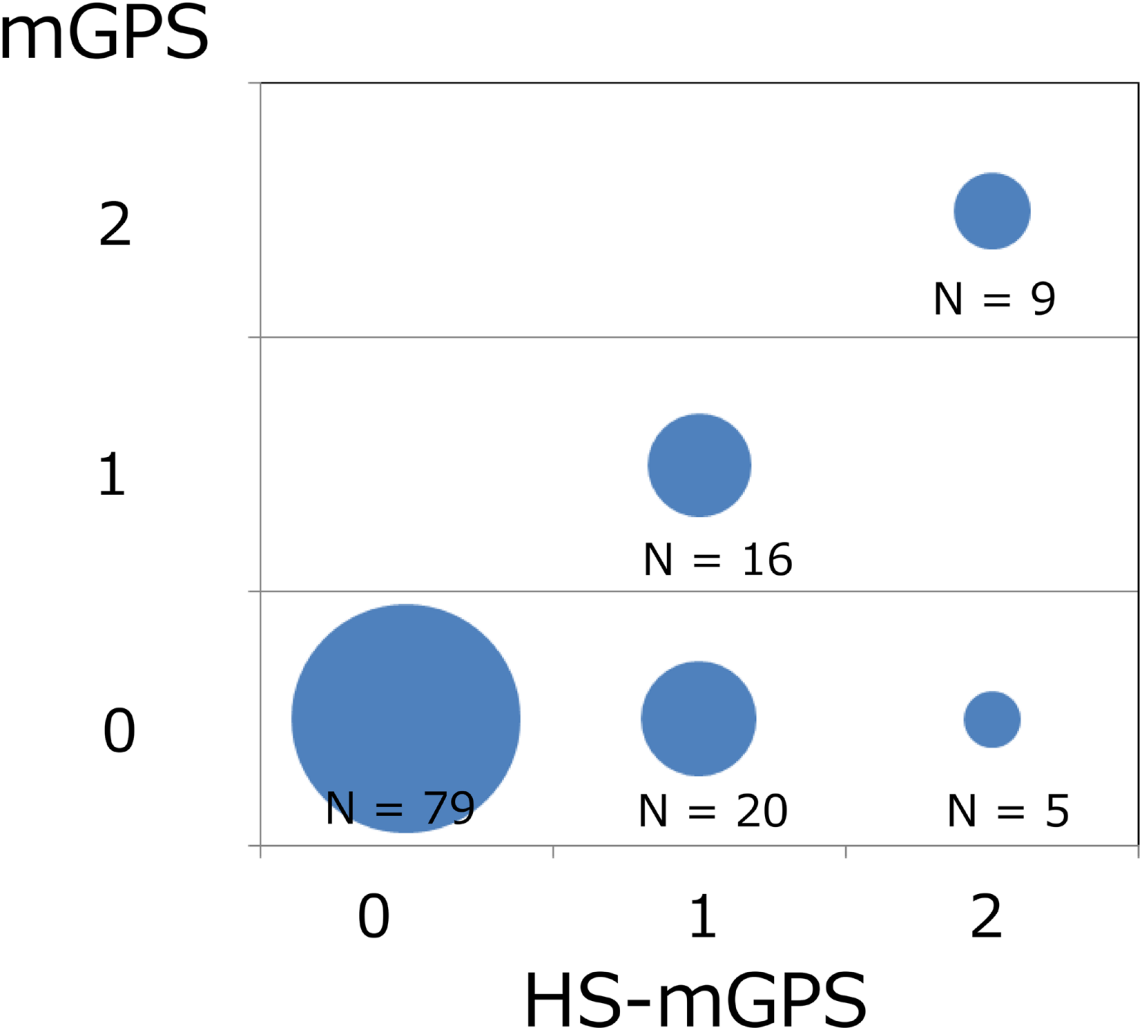
The association between the mGPS and HS-mGPS depicted as a bubble chart Among the 104 patients with an mGPS of 0, 25 were re-classified with an HS-mGPS of 1 (n=20) or 2 (n=5), respectively.

The mGPS classifications of the patients were as follows: 0 (n=104; 80.6%), 1 (n=16; 12.4%) and 2 (n=9; 7.0%). Of the 104 patients with an mGPS of 0, 25 were re-classified as having an HS-mGPS of 1 (n=20) or 2 (n=5) (Figure 1). Among the patients with re-classification, 21 (85%) had Union for International Cancer Control (UICC) stage IV disease; however, no other specific clinical features were observed. The sex and PS were significantly correlated with the mGPS, while the PS and stage were significantly correlated with the HS-mGPS.

### Impact of the mGPS and HS-mGPS on the OS

**Figure 2:**
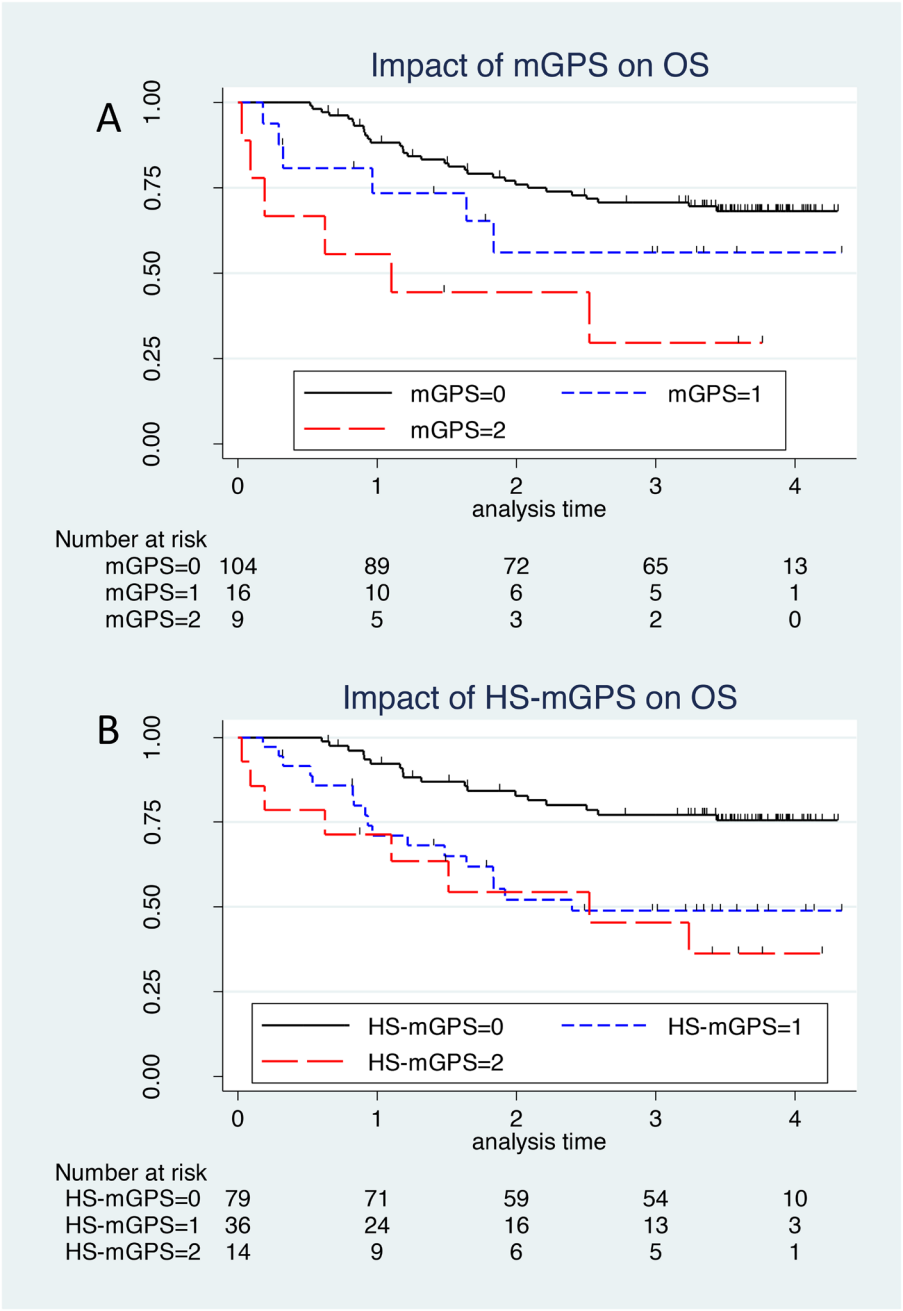
The Kaplan–Meier survival curve of mGPS and HS-mGPS **(A)** The mGPS is statistically significantly associated with the OS (p = 0.003, log-rank test). After adjusting for confounding factor, an mGPS of 2 was associated with a poorer prognosis than that of 0 (adjusted HR comparing mGPS of 2 with that of 0: 2.37 [95% CI, 0.89-6.33], p = 0.084); however, no significant dose-response relationship was observed (trend p = 0.145). **(B)** An elevated HS-mGPS was significantly associated with a poorer survival on a univariate analysis than a reduced score(p = 0.001, log-rank test). Even after adjusting for clinical confounders, the significant association between a higher HS-mGPS and a poorer survival persisted (HR: 2.34 [95% CI: 1.06-5.17], p = 0.035 for HS-mGPS of 1; and HR: 3.14 [95% CI; 1.23-8.07], p = 0.017 for HS-mGPS of 2, compared to HS-mGPS of 0).

Table 3 shows the association between the mGPS/HS-mGPS and the survival. The mGPS was statistically significantly associated with the OS (p = 0.003, log-rank test; Figure 2A). After adjusting for confounding factor, an mGPS of 2 was associated with a poorer prognosis than that of 0 (adjusted HR comparing mGPS of 2 with that of 0: 2.37 [95% CI, 0.89-6.33], p = 0.084; Table 3). However, no significant dose-response relationship was observed (trend p = 0.145).

**Table 3:** Impact of mGPS and HS-mGPS on overall-survival

	N	event	3 years value^2^	95% CI		Univariate analysis				Multivariate analysis^1^			
						HR	95% CI		p-values	HR	95% CI		p-values
mGPS													
0	104	31	0.71	(0.61	-0.79)	1 (reference)				1 (reference)			
1	16	10	0.56	(0.26	-0.78)	1.77	(0.74	-4.25)	0.203	1.01	(0.39	-2.65)	0.980
2	9	3	0.30	(0.05	-0.61)	3.94	(1.64	-9.47)	0.002	2.37	(0.89	-6.33)	0.084
							trend p =		0.002		trend p =		0.145
HS-mGPS													
0	79	61	0.77	(0.66	-0.85)	1 (reference)				1 (reference)			
1	36	19	0.49	(0.31	-0.65)	2.80	(1.44	-5.44)	0.002	2.34	(1.06	-5.17)	0.035
2	14	6	0.45	(0.18	-0.70)	3.78	(1.64	-8.70)	0.002	3.14	(1.23	-8.07)	0.017
							trend p =		<0.001		trend p =		<0.001

1)Adjusted by age, sex, performance status, stage, primary tumor site. mGPS, modifoed Glasgow prognostic score; HS-mGPS, High-sensitivity modified Glasgow prognostic score.

2)3 years = 1080 days.

Regarding the HS-mGPS, an elevated HS-mGPS was significantly associated with a poorer survival on a univariate analysis than a reduced score(p = 0.001, log-rank test; Figure 2B). Even after adjusting for clinical confounders, the significant association between a higher HS-mGPS and a poorer survival persisted (HR: 2.34 [95% CI: 1.06-5.17], p = 0.035 for HS-mGPS of 1; and HR: 3.14 [95% CI; 1.23-8.07], p = 0.017 for HS-mGPS of 2, compared to HS-mGPS of 0; Table 3). In addition, a significant dose-response relationship between the HS-mGPS and OS was observed (trend p < 0.001).

### Impact of the mGPS and HS-mGPS on the OS stratified by variables

**Figure 3:**
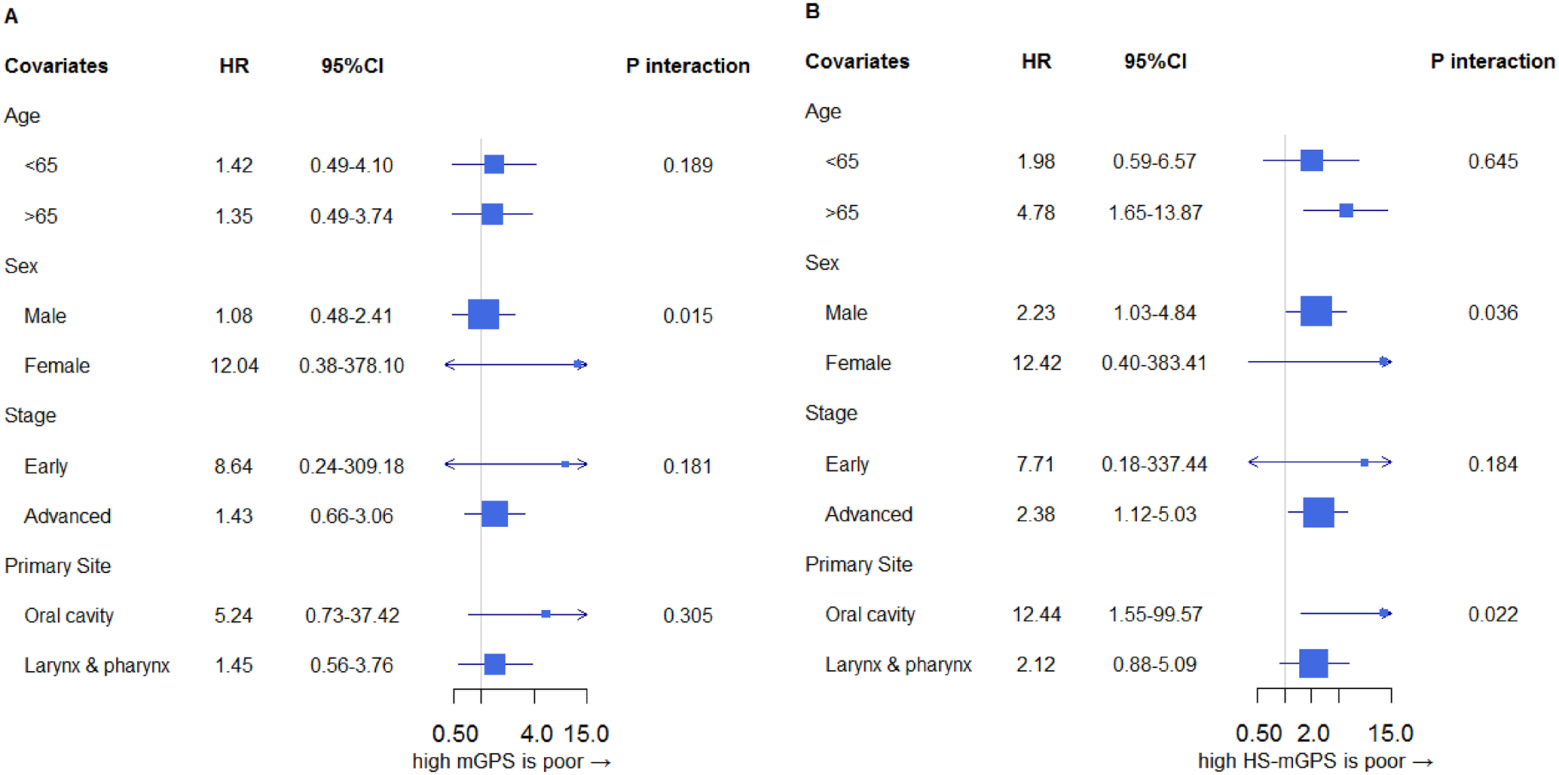
Impact of mGPS **(A)** and HS-mGPS **(B)** on the OS stratified by clinical confounders. **(A)** Compared to an mGPS of 0, an mGPS of 1-2 showed a higher adjusted HR for death in several subgroups; however, statistical significance was not observed in any subgroup. **(B)** A higher HS-mGPS consistently showed increased HRs across all subgroups. The HR of a higher score (score of 1-2) versus a low score (score of 0) was consistently higher in the HS-mGPS **(B)** than in the mGPS **(A)** across almost all subgroups, except for in the early stage. HR: hazard ratio for death of mGPS/HS-mGPS of 1-2 compared to mGPS/HS-mGPS of 0. Adjusted for age, sex, stage, PS, primary site.

After stratification by dichotomized clinical confounders, compared to an mGPS of 0, an mGPS of 1-2 showed a higher adjusted HR for death in several subgroups; however, statistical significance was not observed in any subgroup (Figure 3A).

In contrast, a higher HS-mGPS consistently showed increased HRs across all subgroups (Figure 3B). In particular, among elderly patients, those with oral cavity cancer and women, the prognosis associated with an HS-mGPS of 1-2 was significantly worse than that with an HS-mGPS of 0. In addition, a higher HS-mGPS showed a consistently increased HR across all subgroups except for the early stage groups.

## DISCUSSION

In the present study, we showed that both mGPS and HS-mGPS were significantly associated with the prognosis of HNC in a univariate analysis. After adjusting for clinical covariates, the mGPS was suggestively related to the survival, while the HS-mGPS was significantly associated with the prognosis. The results from the present study indicate that, in terms of the independent prognostic ability, the HS-mGPS may be superior to the mGPS in cases of HNC. This is the first study to evaluate the prognostic impact of both the mGPS and HS-mGPS simultaneously in HNC.

Several studies have investigated the association between mGPS and HNC. In 2010, Proctor et al. reported the prevalence ratio of each mGPS in HNC in their study, although they did not specifically show the data in relation to the prognosis of HNC [21]. Nakayama et al. reported for the first time the clinical utility of the mGPS in patients with HNC [19]. Kawakita et al. later showed the relationship between inflammatory indices, including the mGPS, and the OS in salivary duct carcinoma, which focused mainly on the CRP level [18]. In addition, many studies involving other cancers have shown that the prognosis worsens as the mGPS increases [5–12]. Evidence from these previous studies seems to be in line with the present results.

Furthermore, several studies have suggested that the recently established HS-mGPS is superior to the mGPS as a prognostic marker in many cancers [13–16]; however, little evidence about such an association in HNC has been made available. In the present study, we showed that the HS-mGPS was an independent prognostic marker in HNC. Both the mGPS and HS-mGPS were associated with the OS in a univariate analysis; however, after adjusting for confounding factors, the mGPS showed a suggestive association with the prognosis, while the HS-mGPS was significantly related to the survival. This association persisted across almost all subgroups. These findings agree with the previous results mentioned above [13–16]. Although the underlying mechanism is unclear, we hypothesize that, with the ability to measure the CRP level with a high degree of accuracy, we might be able to detect populations with a poorer prognosis more accurately. With technological advancements, measuring the CRP levels allows us to evaluate inflammation markers very precisely, even those with relatively small values [22]. In addition, there is increased evidence that a CRP level of 0.3 mg/dl is a significant threshold for predicting the prognosis in both cancer and non-cancer patients [21, 23, 24]. As such, our results might be explainable by the assumption that potential cachexia resulting in a poorer prognosis might exist even in cases demonstrating only slight inflammation. HS-mGPS is an independent prognostic factor which can predict survival, even after adjusting for clinical confounders, and it can also detect cases of potential cachexia. For these reasons, HS-mGPS can be said to be more useful than mGPS in HNSCC. Further investigations are warranted.

A previous report suggested the view that the GPS reflects the cachexia status of cancer patients [3]. As such, our findings that the mGPS/HS-mGPS is associated with the survival in HNC may support the interpretation that chronic inflammation and malnutrition are involved in the essential state of cancer cachexia, resulting in a poorer prognosis. In addition, our findings of an increased prevalence of an mGPS/HS-mGPS of 2 even in patients with a PS of 0 suggest that even patients with a good PS can have cachexia. Furthermore, the presence of an mGPS/HS-mGPS of 1 among stage I/II cancer patients suggests that pre-cachexia might be present in patients with an early clinical stage of disease. That several patients with an HS-mGPS of 1 and 2 had previously been classified as having a score of 0 on the mGPS suggests that patients with possible cachexia or pre-cachexia might be misidentified as not having such findings, implying the need for nutritional support even in early-stage HNC patients or those with a good PS. Taken together, these findings suggest that the mGPS/HS-mGPS might be useful indices for making decisions concerning nutritional intervention. Whether or not cachexia can be prevented or treated while still in a reversible state and whether or not the prognosis of cancer patients can be improved by providing nutritional support remain topics for future studies.

In addition, when comparing the prevalence of HS-mGPS/GPS with that of other inflammation markers, such as the neutrophil/lymphocyte ratio (NLR) and platelet/lymphocyte ratio (PLR), an association was thus suggested to exist, with the prevalence of a high NLR/PLR increasing as that of HS-mGPS/mGPS increased (shown in Supplementary Table 1). Further studies will be needed in order to elucidate the association between chronic inflammation and cachexia.

Regarding the primary site specifically, the prevalence of a high-HS-GPS and mGPS in nasal carcinoma seems to be slightly higher than in individuals with cancer at other primary sites. Specific inflammation, such as sinusitis, may therefore be a risk factor for nasal carcinoma, while also suggesting a relationship between malignant tumors and inflammation.

Several strengths associated with the present study warrant mention. First, the eligible participants were selected, in accordance with the inclusion criteria, from among all patients with HNC initially treated at the Department of Head and Neck Surgery of Aichi Cancer Center Central Hospital (ACCH). This reduces the likelihood of selection bias. Second, the clinicians making the treatment decisions in the present study did not determine the mGPS/HS-mGPS, so information bias is also limited.

However, several limitations should also be addressed. For example, this was a retrospective study that was performed at a single institution. The number of cases was limited, and patients who underwent various treatments (e.g. surgery, radiation therapy, etc.) were included in the population. Originally, HNC was included along with cancers of multiple organs, and multidisciplinary therapy with combined modalities is typical in such cases. However, if any bias were present, it would lead to non-differential misclassification, as the treatment would be performed regardless of the mGPS/HS-mGPS. Therefore, this may negate any potential bias in this respect. Furthermore, considering the treatment background might result in more interesting and detailed results. We believe that the results of the present study represent important information directly connected to clinical practices. Therefore, to validate our findings, a larger-scale study incorporating the treatment modality should be performed. In the present study, we excluded MCP as a clinical confounder; however, even after adjusting for MCP, our results remained consistent with those including MCP. (data not shown).

The present study showed that, in terms of the independent prognostic ability, the HS-mGPS might be superior to the mGPS in cases of HNC.

## MATERIALS AND METHODS

### Patient selection

This is a retrospective cohort study evaluating the influence of mGPS and HS-mGPS on the survival among patients with head and neck squamous cell carcinoma. Patients were selected from among participants who underwent initial treatment at the Department of Head and Neck Surgery in ACCH from April 2012 to June 2013. Other inclusion criteria were as follows: 1) primary HNC in the nasal cavity, oral cavity, oropharynx, hypopharynx, or larynx (primary unknown neck metastases, nasopharynx cancer and cervical esophageal cancer were excluded); 2) no history of treatment for HNC (MPC, identified within 6 months of primary head and neck cancer diagnosis was included); 3) pathologically diagnosed as squamous cell carcinoma; and 4) cases in which the CRP and albumin levels had been measured at the initial diagnosis.

In the present study, a total of 230 consecutive patients initially treated at our department were eligible. Of these, in accordance with criteria, 129 patients with head and neck squamous cell carcinoma were included in the analysis. All participants provided their written informed consent to participate.

### Statistical analyses

In the present study, the mGPS and HS-mGPS were the main exposures of interest. We calculated these values using the data from the blood examination at the first visit. Regarding the mGPS, patients were classified as follows [6, 20]: patients with both an elevated CRP level (>1.0 mg/dl) and reduced albumin level (<3.5 g/dl) were given a score of 2; those with an elevated CRP level (>1.0 mg/dl) and a non-decreased albumin level (≥3.5 g/dl) were given a score of 1; and those with a non-elevated CRP level (≤1.0 mg/dl), regardless of their albumin level, were given a score of 0. The modified BCP assay was used to measure the serum albumin level in this study. With regards to the HS-mGPS, the cut-off CRP level was 0.3 mg/dl, as reported by Proctor et al. [15] (Table 1).

The primary endpoint of this study was the overall survival (OS; the interval between the date of the first visit [same date as the blood examination] and the date of death from any cause or the date of last follow-up), estimated by the Kaplan–Meier method. Participants who were lost to follow-up were treated as censored. To evaluate the survival impact of the mGPS and HS-mGPS, we estimated the hazard ratio (HR) and 95% confidence interval (95% CI) using multivariate Cox proportional hazards models.

Confounding variables considered in the multivariate analyses were age (<65 vs. ≥65 years), sex (male vs. female), Eastern Cooperative Oncology Group performance status (ECOG PS: 0, 1, 2), clinical stage (UICC 7^th^ edition: 1, 2, 3, 4) and primary tumor site (nasal cavity, oral cavity, oropharynx, hypopharynx and larynx). Stratification was performed by dichotomized confounding variables. All statistical analyses were performed using the JMP software program (version 13.0.0; SAS Institute Inc., USA). All tests were 2-sided, and P-values <0.05 were considered statistically significant.

## CONCLUSIONS

We showed that the mGPS/HS-mGPS have prognostic utility in patients with HNC. In addition, the HS-mGPS may be a more sensitive index than the mGPS and was found to be an independent prognostic factor.

## SUPPLEMENTARY MATERIALS TABLE


