# Supplementary material for: The high-sensitivity modified Glasgow prognostic score is superior to the modified Glasgow prognostic score as a prognostic predictor for head and neck cancer
Source: Oncotarget. 2018 Dec 11;9(97):37008–16. doi: 10.18632/oncotarget.26438 (PMC6319335; doi:10.18632/oncotarget.26438)
Supplement: Supplementary file 1 [file oncotarget-09-37008-s001.pdf]

The high-sensitivity modified Glasgow prognostic score is superior to the modified Glasgow prognostic score as a prognostic predictor for head and neck cancer

SUPPLEMENTARY MATERIALS

Supplementary Table 1: The association between HS-mGPS/mGPS and NLR/PLR

**Characteristics**			**HS-mGPS**										**mGPS**								
			0			1			2		**p-value†**		0			1			2		**p-value**
	N (%)		*(N =* 79)	*(%)*		*(N =* 36)	*(%)*		*(N =* 14)	*(%)*			*(N =* 104)			*(N =* 16)			*(N =* 9)		
NLR‡,																					
Continuous (mean [± SD])	80 (62)		3.16 (± 1.69)			4.81 (± 3.16)			8.60 (± 10.14)				3.53 (± 2.11)			5.55 (± 3.52)			9.98 (± 11.50)		
Dichotomized††																					
Low (<3.5)	44 (34)		28	(35)		12	(33)		4	(29)	0.04		37	(36)		4	(25)		3	(33)	0.115
High (≥3.5)	36 (27)		13	(16)		15	(42)		8	(57)			23	(22)		7	(44)		6	(67)	
missing	49 (38)		38	(48)		9	(25)		2	(14)			44	(42)		5	(31)		0	(0)	
PLR§,																					
Continuous (mean [± SD])			171 (± 133)			207 (± 115)			*269* (± 170)				178 (± 125)			227 (± 118)			292 (±187)		
Dichotomized††																					
Low (<193)	33 (26)		34	(43)		13	(36)		5	(36)	0.002		45	(43)		4	(25)		3	(33)	0.005
High (≥193)	35 (27)		7	(9)		14	(39)		7	(50)			15	(14)		7	(44)		6	(67)	
missing	49 (38)		38	(48)		9	(25)		2	(14)			44	(42)		5	(31)		0	(0)	

† chi-square test, excluding missing value status
‡ NLR: neutrophil/lymphocyte ratio
§ PLR: platelet/lymphocyte ratio
¶ The NLR/PLR of 33 patients was collected within 7 days from the date of the GPS examination
†† The cut-off point of those scores was estimated based on the receiver operating characteristics (ROC) curve with death within two years as the defining point
